# Cardiac Autonomic Dysfunction in Patients with Schizophrenia and Their Healthy Relatives – A Small Review

**DOI:** 10.3389/fneur.2015.00139

**Published:** 2015-06-24

**Authors:** Karl-Jürgen Bär

**Affiliations:** ^1^Psychiatric Brain and Body Research Group Jena, Department of Psychiatry and Psychotherapy, University Hospital, Friedrich-Schiller-University, Jena, Germany

**Keywords:** continuous training, heart rate variability, exercise intervention, sudden cardiac death, autonomic dysfunction

## Abstract

The majority of excess mortality among people with schizophrenia seems to be caused by cardiovascular complications, and in particular, coronary heart disease. In addition, the prevalence of heart failure and arrhythmias is increased in this population. Reduced efferent vagal activity, which has been consistently described in these patients and their healthy first-degree relatives, might be one important mechanism contributing to their increased cardiac mortality. A decrease in heart rate variability and complexity was often shown in unmedicated patients when compared to healthy controls. In addition, faster breathing rates, accompanied by shallow breathing, seem to influence autonomic cardiac functioning in acute unmedicated patients substantially. Moreover, low-physical fitness is a further and independent cardiac risk factor present in this patient population. Interestingly, new studies describe chronotropic incompetence during physical exercise as an important additional risk factor in patients with schizophrenia. Some studies report a correlation of the autonomic imbalance with the degree of positive symptoms (i.e., delusions) and some with the duration of disease. The main body of psychiatric research is focused on mental aspects of the disease, thereby neglecting obvious physical health needs of these patients. Here, a joint effort is needed to design interventional strategies in everyday clinical settings to improve physical health and quality of life.

## Background

A large body of evidence has documented shortened life expectancy in patients with schizophrenia (1–4). It has been assumed that suicides, accidents, and cardiovascular disorders are the main reasons for the excess of premature and sudden deaths among patients with schizophrenia (3–6). In patients treated with antipsychotics, research showed evidence that the incidence-rate ratio of sudden cardiac death (SCD) was doubled in individuals receiving first- or second-generation antipsychotics in the last month of life (7). The dose-dependent effect of antipsychotics on myocardial cell repolarization was assumed to lead to torsades de pointes, arrhythmias, and, finally, to ventricular fibrillation and SCD. In this line of evidence, a recent study reporting autopsy findings in inpatients with schizophrenia showed that cardiovascular disorders were the most common cause of death (8). Thus, schizophrenia may represent a disorder with a specific cardiac vulnerability to SCD (9). This assumption is supported by a recent study by Mothi et al. (10) showing that cardiovascular and metabolic dysfunction is increased in healthy first-degree relatives of patients. This is very suggestive of an overlapping genetic background of cardiac/metabolic conditions and psychotic disorders.

Sudden cardiac death happens when a malignant arrhythmia is triggered by an acute cardiac event (e.g., acute myocardial ischemia, platelet activation, or neuroendocrine variations) on the basis of a diseased myocardium (e.g., post-necrotic scar or hypertrophy). In addition to coronary artery disease or diseases of the myocardium, cardiac electrophysiological abnormalities might predispose to the development of ventricular fibrillation. This is especially important after acute myocardial infarction (AMI). Physicians found that various indices of heart rate variability (HRV) are of predictive value for the outcome of patients after AMI. Subsequently, these measures were transferred to other patient populations. Abnormalities were found for patients suffering from depression, anxiety disorders, alcohol dependence, and, in particular, patients suffering from schizophrenia (11–16). The main difference, however, is the significance of these values. After AMI, the risk prediction of HRV values for SCD is defined by a measurable endpoint (death). By contrast, the exact meaning of reduced HRV measures in mental disorders is more difficult to define, since patients live with the disease and altered cardiac autonomic function for many years. Therefore, the definite influence of profound autonomic dysfunction in patients with schizophrenia for reduced life expectancy needs to be shown in long-term prospective studies.

## Heart Rate Variability

The term HRV refers to a number of measures of different types. In general, nearly all HRV measures reflect mainly vagal (parasympathetic) modulation at the level of the heart. HRV is the physiological phenomenon of variation in the time interval between heart beats and it is determined by measuring the variation in the beat-to-beat interval (BBI). Although HRV measures can be obtained quite easily nowadays, there are numerous pitfalls. Autonomic indices depend very much on circadian rhythms, the duration and measurement procedure, the environment, and the artifact management. Time domain and frequency domain measures are most often used. In general, if time domain measures [e.g., root mean square successive difference (RMSSD)] are extremely low, true autonomic dysfunction can be assumed. Frequency domain measures [e.g., high frequency (HF), low frequency (LF), very low frequency (VLF)], which are very susceptible to artifacts, quantify the amount of variance in heart rate at different underlying frequencies. Again, extremely low values of HF are associated with a lack of autonomic vagal modulation of heart rate.

Besides linear HRV parameters describing the variance of BBIs, non-linear complexity measures have been developed to describe the regularity of heart rate time series. The application of these novel analyses has led to a higher sensitivity for detecting autonomic dysfunction (17, 18) and patients at risk for sudden death (19). A high complexity of biosignals reflects diverse influences of different regulatory systems. In the case of BBI, these are, among others, neuronal (autonomic nervous system), hormonal (e.g., cortisol, ANP), and myocardium inherent mechanisms (20). Overall, up to a certain point, the more irregular and complex heart rate series are the more adaptive and stable is the underlying system. Such complexity is only in part reflected in measures of classical moment statistics such as means and SEs (e.g., time domain or frequency domain measures), which mainly describe the fluctuation of the biosignal. However, classical parameters do not contain sufficient information on the regularity pattern of these fluctuations. For example, a sine curve might have the same mean and SE as a very irregularly shaped curve, which is why non-linear measures are required to detect these differences in system complexity.

## Heart Rates of Unmedicated Patients Suffering from Schizophrenia

“Taking the pulse” has always been the first point of contact between physicians and the patient. It has recently been suggested that the heart rate corresponds to the rate of energy needed by the body. A reduction in heart rate of 10 bpm a day saves 5 kg of adenosintriphosphate (ATP). Furthermore, an increase in heart rate of 5 bpm corresponds to a significant increase in the atherosclerosis progression. Animal and human studies show that life expectancy is closely related to the medium heart rate. Increased resting heart rate has been shown to be a risk factor for reduced life expectancy in both the general population (21, 22) and in populations with cardiovascular diseases (23, 24).

In 1899, Emil Kraepelin described extensive autonomic alterations in patients with schizophrenia, including increased heart rates, altered pupillary function, increased sweating and salivation, as well as temperature changes (25). Most of these described signs suggest increased sympathetic output, decreased parasympathetic modulation, or both. For a long time, psychiatrists attributed increased heart rates in patients with schizophrenia to antipsychotic treatment. This assumption is only to some extent correct. Treatment with clozapine, for instance, is associated with reduced vagal function and increased heart rates (26–28). However, several studies have reported increased heart rates in first episode and unmedicated patients (26, 29–31). In a pooled analysis, we found that among 119 unmedicated patients the heart rate at rest was increased by about 10 bpm in over 40% of patients and by about 20 bpm in 25% of patients (unpublished data). It is important to understand that antipsychotic drugs might increase heart rates due to anticholinergic side effects even further (32). Here, a dose-dependent increase has been described (28). However, other authors have found improved autonomic function after the introduction of antipsychotic treatment, possibly due to changes in clinical presentation (33) or only minor effects of treatment on cardiac autonomic function (34).

**Figure 1:**
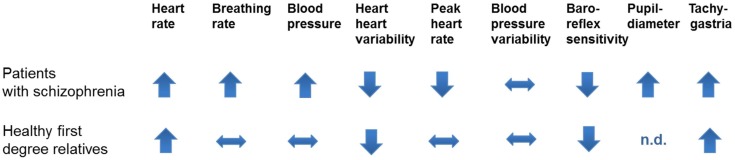
**The figure shows investigated autonomic domains in patients with schizophrenia and their healthy first-degree relatives**. Peak heart rates indicate heart rates during vigorous exercises. Tachygastria is a sympathetic parameter obtained in the electrogastrogramm.

Investigations in healthy first-degree relatives of patients displayed similarly increased heart rates although less pronounced (35–39). Comparable to other findings such as structural brain changes in healthy relatives of patients with schizophrenia (40) autonomic dysfunction seems to have a genetic basis. A summary of investigates autonomic domains in patients and their healthy relatives is shown in Figure 1.

## Time and Frequency Domain Parameters of HRV in Patients with Schizophrenia

As described above, HRV is the beat-to-beat oscillation of RR intervals around its mean value. It is the result of complex regulatory mechanisms through which the autonomic nervous system influences heart rate and keeps cardiovascular parameters within physiological health ranges.

Both time and frequency domain parameters of HRV show reduced efferent vagal activity in unmedicated patients (29, 32, 36, 41–44). Described results were obtained by short time measurements (5 and 30 min) as well as by 24-h holter ECG measurements. This effect cannot be explained by increased heart rates alone, nor is it solely allegeable by sympathetic modulation.

## Complexity Measures of Heart Rate in Patients with Schizophrenia

A concept that is closely connected to that of variability is complexity. It is important to realize that complexity is different from variability and there is no simple definition for it. A time series might show high variability and very low complexity. “Simple” time series are readily understood, and can be described concisely as having low information content or low complexity. Conversely, complex series are not easy to understand completely, they are full of unforeseeable shifting and require lengthy descriptions, making their information content high. Complex signals produced by healthy organisms might have dynamic properties such as non-linearity (the relationships among components are not additive, so small perturbations can cause large effects) or non-stationarity (statistical properties of the system’s output change with time). Thus, high complexity describes to a certain degree healthy physiological properties, while low complexity describes reduced influence of various regulatory circuits. Vice versa, the more irregular and complex heart rate series are the more adaptive and stable is the underlying system. Such complexity is only in part reflected in measures of classical moment statistics such as means and SEs, which mainly describe the fluctuation of a biosignal (45). Since no single measure is sufficient to capture the properties of the most complex signals, different complexity measures are needed. For heart rate in patients with schizophrenia, we have mainly used compression entropy (17), measures of symbolic dynamics (19, 46), and approximate entropy (47).

To give an example of the calculation of complexity measures, compression entropy (Hc) is described below: the entropy (complexity) of a given text is defined in terms of the smallest algorithm that is capable of generating the text. Although it is theoretically impossible to develop such an algorithm, data compressors represent a sufficient approximation. The LZ77 algorithm for loss-less data compression introduced by Ziv and Lempel (48) is widely used and implemented in many file compressors such as Winzip^®^. Its application in RR time series has been described by Baumert et al. (17). The ratio of the compressed to the original time series length represents an index of entropy and is referred to as compression entropy Hc. Thus, compression entropy indicates the degree to which data from heart rate time series can be compressed using the detection of recurring sequences. The more frequent certain sequences occur – and therefore, the more regular these series are – the higher the compression rate.

Similarly to variability parameters, various studies during the last years have described reduced complexity of heart rate dynamics in patients with schizophrenia (32, 42, 49–52). The reduction in complexity indicates that in patients with schizophrenia, the heart rate cannot adapt to different requirements arising from posture or exertion and that the heart is at higher risk of developing arrhythmias. In addition, one can speculate that reduced regulatory influence from the vagal system might contribute to reduced complexity. It is a method-inherent problem that complexity measures cannot be attributed to one single physiological system.

## Baroreflex Sensitivity

The evaluation of baroreflex sensitivity (BRS) is an established tool for the assessment of autonomic control of the cardiovascular system. The baroreflex or baroreceptor reflex is one of the body’s homeostatic mechanisms to maintain blood pressure at nearly constant levels. The baroreflex provides a negative feedback loop in which an elevated blood pressure reflexively causes the heart rate to decrease. By contrast, diminished blood pressure reduces baroreflex activation and causes heart rate to increase. A reduction in baroreflex control of heart rate has been reported in hypertension, coronary artery disease, myocardial infarction, and heart failure (53). There are various methods to assess BRS. For patients with schizophrenia, the non-invasive sequence method is used (54). Here, spontaneous sequences of at least three consecutive beats are analyzed, when an increased systolic blood pressure (SBP) of at least 1 mmHg causes an increased BBI of at least 5 ms (bradycardic sequence) or a decreased SBP causes a decreased BBI (tachycardic sequence). For each sequence, the regression between the three SBP values and three BBI values is calculated and the slope (tachycardic slope: tslope; bradycardic slope: bslope) of the regression line is used as an index of BRS.

Studies in unmedicated patients with schizophrenia show significantly reduced tachycardic and bradycardic slopes (31, 35, 55, 56). Thus, the fine-tuning of blood pressure and heart rate is severely impaired among acute psychotic patients. Interestingly, blood pressure values and blood pressure variability are only marginally altered in these patients (57). We have therefore concluded that the primary change in patients is observed in the heart rate domain. To explain putative mechanisms for reduced BRS in patients, it is important to realize that powerful negative feedback loops between heart rate and blood pressure can be inhibited to allow the organism to adjust to demanding environmental strains (inhibition of baroreflex vagal bradycardia, BVB). Thus, BRS has been shown to decrease during specific cognitive demands, such as basic arithmetic operations (58) or physical activity (59). Thus, this might imply that the decrease of efferent vagal activity and the inhibition of BVB in acute schizophrenia are actually caused by stress due to psychotic experiences or the psychosis itself, a process that allows the organism under physiological conditions to adjust to demanding environmental strains.

## Patients Breathing Rates

During acute episodes, a putative relation between breathing rates and symptom severity were described in patients with schizophrenia 80 years ago (38, 60–63). The German Psychiatrist Wittkower described the breathing rate in psychotic patients to be faster and more regular. In our analysis, we found that a fast breathing rate is the dominant feature in unmedicated patients and that it is accompanied by some shallowness of breathing. We also found more variability within the breathing pattern (38). However, the minute ventilation is not altered in patients. Interestingly, when healthy subjects breathe in the modus of patients, we observed increased heart rates and reduced variability (unpublished data). Healthy relatives of patients do not show changes within their breathing pattern (38). We speculate that the breathing pattern is closely associated with symptoms during acute episodes, while the HRV pattern seems to be a trait marker. Of course, it is impossible to disentangle breathing and HRV completely because of their close interrelationship.

## Exercise and Autonomic Function

Cardiorespiratory fitness is a strong and independent mortality predictor for humans (64). Therefore, it is important to investigate fitness in patients with schizophrenia, since it might be one approach for modifying the increased cardiac mortality risk associated with the disease. Overall, reduced physical fitness is a commonly reported trait among patients with schizophrenia that can be improved by means of physical interventional studies (16, 65, 66). Ostermann et al. (67) investigated autonomic function during physical exercise. Interestingly, they showed increased breathing rates and reduced vagal modulation during the entire test. However, heart rates were only initially increased in comparison to controls. The authors reported that reduced vagal function during the exercise test correlated with the inflammatory response after exercise as assessed by TNFalpha levels. This result touches on a further important relationship between vagal modulation and inflammatory response (68). Most interestingly, in a new study, Herbsleb et al. (16) show that chronotropic incompetence (CI), which is a strong predictor for cardiovascular mortality, is reported in about 60% of patients with schizophrenia taking regular medication. CI is defined as the inability of the heart to increase its rate commensurate with increased activity or demand. It has been established as a predictor of cardiovascular events and all-cause mortality (69). Most interestingly, the authors describe similarly a lack of catecholamine increase and a close correlation between CI and the duration of disease. Thus, future studies need to investigate the cardiovascular benefit, which patients might gain due to different types of exercise to reduce their potential cardiovascular risk profile (16).

## Psychopathology and Autonomic Function

Autonomic dysfunction is most likely the consequence of long-lasting stressful experiences associated with the psychotic state, in addition to a genetic underlying predisposition to autonomic dysfunction as observed in relatives of patients. Therefore, the notion of a relation between the severity of the disease assessed by the global assessment of functioning scale (GAF) and autonomic dysfunction is not surprising (70). However, it is rather interesting that autonomic dysfunction seems to be somehow related to the degree and amount of delusional states found in patients (29, 49, 55, 56). A clear relation to negative symptoms was less often observed (42). Altogether, there is no simple and linear relation between the severity of a current episode and the degree of autonomic dysfunction but some relation to the delusional state.

## Future Perspectives

There are three important areas for future research in patients with schizophrenia. First of all, studies need to investigate the definite relation between the degree of autonomic dysfunction and the potential risk of cardiovascular events for these patients. At a minimum, schizophrenic patients at increased risk should be identified. Second, the brain activations underlying autonomic dysfunction need to be assessed to elucidate pathophysiological mechanisms. Third, psychiatric research is focused mainly on mental aspects of the disease, thereby neglecting obvious physical health needs of patients with schizophrenia. Here, a joint effort is needed to design interventional strategies in everyday clinical settings to improve physical health and quality of life of our patients.

## Conflict of Interest Statement

The author declares that the research was conducted in the absence of any commercial or financial relationships that could be construed as a potential conflict of interest.
